# Behavioral Methods for Severity Assessment

**DOI:** 10.3390/ani10071136

**Published:** 2020-07-03

**Authors:** Pia Kahnau, Anne Habedank, Kai Diederich, Lars Lewejohann

**Affiliations:** 1 German Federal Institute for Risk Assessment (BfR), German Center for the Protection of Laboratory Animals (Bf3R), 12277 Berlin, Germany; Anne.Habedank@bfr.bund.de (A.H.); Kai.Diederich@bfr.bund.de (K.D.); Lars.Lewejohann@bfr.bund.de (L.L.); 2 Institute of Animal Welfare, Animal Behavior and Laboratory Animal Science, Freie Universität Berlin, 14163 Berlin, Germany

**Keywords:** severity assessment, animal welfare, refinement, preference test, cognitive bias

## Abstract

**Simple Summary:**

In 2017, 9.4 million animals were used for research and testing in the European Union. Animal testing always entails the potential for harm caused to the animals. In order to minimize animal suffering, it is of ethical and scientific interest to have a research-based severity assessment of animal experiments. In the past, many methods have been developed to investigate animal suffering. Initially, the focus was on physiological parameters, such as body weight or glucocorticoids as an indicator of stress. In addition, the animals’ behavior has come more into focus and has been included as an indicator of severity. However, in order to obtain a comprehensive understanding of animal suffering, an animal’s individual perspective should also be taken into account. Preference tests might be used, for example, to “ask” animals what they prefer, and providing such goods in turn allows, among other things, to improve housing conditions. In this review, different methods are introduced, which can be used to investigate and evaluate animal suffering and well-being with a special focus on animal-centric strategies.

**Abstract:**

It has become mandatory for the application for allowance of animal experimentation to rate the severity of the experimental procedures. In order to minimize suffering related to animal experimentation it is therefore crucial to develop appropriate methods for the assessment of animal suffering. Physiological parameters such as hormones or body weight are used to assess stress in laboratory animals. However, such physiological parameters alone are often difficult to interpret and leave a wide scope for interpretation. More recently, behavior, feelings and emotions have come increasingly into the focus of welfare research. Tests like preference tests or cognitive bias tests give insight on how animals evaluate certain situations or objects, how they feel and what their emotional state is. These methods should be combined in order to obtain a comprehensive understanding of the well-being of laboratory animals.

## 1. Introduction

In 2017, 9.4 million animals were used for research and testing purposes in the European Union. Mice were the most commonly used experimental animal species (61%), followed by fish (13%) and rats (12%) [1]. These animals were used either in basic research or translational and applied research but also for regulatory use and routine manufacture of medical products [1]. It is acknowledged that all animal research shall be conducted under the premise of the 3Rs (Reduce, Replace, Refine) according to Russell and Burch [2]. In light of the longstanding debate on the ethical acceptability of animal experiments, it is a moral imperative that all experiments, regardless of the species used, be double-checked for opportunities to use alternative methods. In addition, only as few animals as absolutely necessary shall be used. Finally, all animal research that cannot be reduced or replaced must seek the best possible refinement to be ethically acceptable. In order to minimize the burden laid on animals, it is of ethical and scientific interest to have valid methods for determining animal suffering in animal experiments. Furthermore, and this is important to note, the suffering of the animals can have a profound negative impact on the experimental data. Only if the extent of the suffering is known it is possible to use this information to both strengthen animal welfare and improve the results and validity of future experiments. Animal welfare measures the status of a subjectively perceived quality of life of an individual and is notably hard to access and disentangle [3]. Thereby, animal welfare comprises various aspects, such as animal life quality, health status, biological function, and subjective feelings [4,5,6,7]. Apart from objectively measurable deterioration, animal welfare is also affected by the capacity of animals to cope with environmental challenges [8]. Overall, various factors such as social interaction, housing conditions, human handling or laboratory procedures affect animal welfare [9]. It is noteworthy that these different factors can simultaneously influence animal welfare in a non-linear way: Although positive social interaction does not directly influence the perception of pain, it can improve the overall welfare of, e.g., injured animals [3]. All this has to be taken into account for assessing the severity of procedures as well as the potential refinement measures for eliciting positive affective states [10].

In the European Union Directive 2010/63/EU, Article 38, 39, 54 and Annex VIII it is specified that all procedures involving laboratory animals have to be classified into one of four categories describing the severity of the procedure. These categories are “mild”, “moderate”, “severe” and “non-recovery” [11]. In the European Union in 2017, 51% of all procedures using animals in research and testing were classified as “mild”, 32% were classified as “moderate”, 11% as “severe” and 6% as “non-recovery” [1]. While “non-recovery” naturally means damage to the animal, paradoxically there is little concern here for the welfare of the animals, since with the death of the animal the capacity for suffering itself is also ended. However, experiments classified in any of the other three categories are under scrutiny regarding the severity of the conditions imposed on the animals so that the defined limits are not exceeded. For the classification of animal suffering, score sheets are used to assess pain, suffering or harm during animal experiments. In planning an animal experiment, all expected burdens have to be defined within these score sheets along with all measures which will be taken to reduce animal suffering. Score sheets should be efficient, easy to follow and adapted to the specific experiment. In addition, researchers and caretakers using score sheets should be well trained to unequivocally recognize and score any changes in animal welfare [12]. Ullmann and colleagues outlined recommendations for the preparation and usage of such score sheets [13]. The score sheets shall include all experiment-specific considerations, for example, van de Meer and colleagues created a score sheet for severity assessment of transgenic mice [14], and Lang and colleagues for osteotomy models in rats and mice [15]. Rix and colleagues used a score sheet for mice, which were given various chemotherapeutic agents, to study the applicability of this score sheet [16]. Only changes in body weight indicated a change in well-being of mice. Since body weight reduction could also be a side effect of chemotherapy the authors suggested to improve score sheets for experiments with chemotherapy trials by including behaviors such as nausea and fatigue into the scoring [16].

Indications of animal suffering can be derived from physiological parameters. Some studies showed that the body weight decreased during distress [17,18,19]. Rats which were restrained on three consecutive days showed a decreased food intake leading to a decreased body weight compared to non-restrained rats. This reduction was eminent for over 40 days after restraining [18]. However, it should be noted that body weight can be influenced, for example, by tumor growth or fluid accumulation, thus possibly masking any stress-related body weight reduction [20].

Other physiological stress parameters are glucocorticoid stress hormones, which increase in the body as a result of suffering or stress [21]. Glucocorticoids or their metabolites are commonly measured in blood [22], feces [23] or in hair samples [24]. Leenaars and colleagues performed a mapping review to analyze the frequencies of corticosterone sample types in mice and the different analysis techniques [25].

Such physiological parameters could provide indications of changes in well-being. However, the interpretation does not always seem to be easy. For example, factors such as duration and intensity of changes in physiological parameters must be taken into account [3]. It is also important to record the nature of the situations in which these changes occur. Glucocorticoids, for example, also increase in situations that are not considered to be related to suffering such as mating [26]. Nevertheless, a lack of changes does not necessarily mean that the animal has an unchanged well-being [10]. Therefore, it is deemed useful to extend severity assessment to other parameters like behavior, preferences, or the emotional state.

## 2. Including the Animal’s Behavior

Some experiments, for example, those involving surgical procedures or the application of pharmaceuticals, potentially inflict pain and suffering [27,28,29,30,31]. Treatment-induced suffering can be assessed through a comprehensive behavioral observation. Especially comfort-related behaviors such as nesting and burrowing are used to assess the animal’s burden as it is assumed that comfort behaviors decrease in the presence of pain, suffering, or harm [31,32]. Jirkof and colleagues pointed out that nest-building is part of thermoregulation in small rodents, therefore, complex nest-building behavior could be an indication of unfavorable temperature conditions [10]. Häger and colleagues developed a model, in which wheel running was used to assess the severity level for mice in a colitis model. It was shown that the activity in the running wheel is indeed a useful indicator of compromised welfare in mice with a decrease in wheel running associated with increasing severity [19]. Other behaviors like twitching and writhing directly indicate pain [30,33,34]. For example, Roughan and colleagues showed that after surgery, pain behavior was significantly less expressed in rats which were given analgesia compared to rats without analgesia treatment. Based on this knowledge, they developed a pain scoring method for abdominal surgeries [33]. As direct observations are very time consuming and involve the risk of an observer bias, Roughan and colleagues used commercially available software-supported video observations to analyze activity behavior. The software identifies various behaviors such as walking, digging or stretching [35].

Another method for pain assessment is the Grimace Scale, developed first in mice by Langford and colleagues [36]. In this method the facial field of an animal is photographed and evaluated according to certain parameters (e.g., ear position, whiskers, etc.). Overall, this results in a score indicating the level of pain. In other studies, the Grimace Scale was used to assess the effectiveness of analgesics and the influence of repeated anesthesia [24,30,34,37]. The Grimace Scale is a useful method to measure suffering in laboratory animals, although this method is time consuming and there is also the possibility of an observer bias. Therefore, methods are being developed that perform images and video analysis automatically [38,39,40].

Abnormal behaviors such as stereotypies can also be an indication of animal suffering. Stereotypies are constant and repeated sequences of movements that do not seem to have any obvious utility [41], and can be developed under impoverished environmental conditions, but also as a result of fear or frustration [42]. Powell and colleagues showed that deer mice housed under standard conditions developed stereotyped behaviors earlier and in a higher rate compared to deer mice housed under enriched conditions [43]. Stereotypies may indicate poor well-being but for a profound assessment it is important to consider the frequency of stereotypic behavior, the situations when they occur and the individual characteristics of each animal [42,44].

## 3. Preference Tests

The physiological and behavioral parameters outlined above are important indicators of animal suffering. However, there is still a large scope for interpretation from the human perspective. It is therefore necessary to develop methods that include the animal’s perspective in order to gain a more comprehensive understanding of severity assessment and animal welfare.

One such animal-centered method is preference testing. Preference tests allow the animal to choose between different goods for a defined period of time. The good that is selected more frequently or for a longer period of time is considered the preferred one. Preference tests have been used frequently and in different ways [45]. However, it should also be noted that choices can be influenced by previous experiences or the current motivational state of the animal [46,47]. For example, Dawkins showed that hens normally preferred litter-floored cages without food rather than wire-floored cages with food. However, if hens previously had no access to food, the hens preferred the wire-floored cage with food [48].

In order to optimize animal husbandry, preference tests can be used to determine which type of cage design or arrangement animals prefer. Among other things, the amount of bedding provided in the home cage was examined: Freymann and colleagues showed by means of preference tests that a larger amount of bedding is preferred by mice over home cages with less bedding. The authors also showed that mice with a large amount of bedding had lower corticosterone titers than mice with less bedding. However, the behavior (e.g., agonistic behavior, locomotion, nest-building, grooming) did not seem to be influenced by the amount of bedding [49]. The preference test was also utilized to determine preference for enrichment items. Lewejohann and Sachser showed that an enriched cage with hiding and climbing possibilities is preferred by male mice over a standard cage without enrichment items [50]. Banjanin and Mrosovsky examined running wheels made of different materials for rodents and showed that mice had a high preference for plastic mesh flooring over metal rods [51].

The Conditioned Place Preference Test (CPP) is mostly used to investigate the effects of drugs [52,53]. The CPP is based on classical (Pavlovian) conditioning, in which a previously neutral stimulus (conditioned stimulus, e.g., floor pattern or odor) is associated with an event eliciting a motivational response (unconditioned stimulus, e.g., drug vs. vehicle). Conditioning itself takes place by confining the animals alternately to two distinct compartments, of which each contains a different neutral stimulus of the same modality (e.g., a pattern of dots vs. a pattern of stripes). In one compartment, the animal is then also exposed to the unconditioned stimulus. In this manner the neutral stimulus is associated with the unconditioned response, and thus becomes a conditioned stimulus. After conditioning, the previously neutral condition should induce the same response as the unconditioned stimulus [54]. Thereby, preference or avoidance can be assessed without using the unconditioned stimuli themselves in order to avoid direct negative effects or habituation to the stimuli. These findings can also be helpful in evaluating animal experiments associated with pain in relation to animal suffering. For example, the CPP has already been used to examine the effect of analgesic drugs [55,56] and has also been used to show, for example, that fish prefer an appetitive stimulus over being chased with a net [57]. In young mice it has been shown that social proximity is rewarding [58]. However, expanding the CPP to a general animal welfare assessment tool has proven to be difficult because results are easily influenced by additional motivations, e.g., spending time in a more familiar environment, or foraging instead of paying attention to the presented stimuli [59].

In addition, if an animal has made a choice and a preferred good has been determined, this does not necessarily mean that this choice is objectively the best choice for the animal. For example, many animals tend to show a strong preference for saccharin despite the lack of caloric gain, or a preference for alcohol regardless of the negative health consequences. A preference for a certain good also does not necessarily imply that if the animal does not have access to this preferred good that the animal will suffer [60]. This is especially true for luxury items or goods that can be easily surrogated by alternative goods. Therefore, it is reasonable to examine the quality of the tested goods more closely, for example, by using the consumer demand test.

## 4. Consumer Demand

Consumer demand tests can be used to determine the strength of preference for a preferred good. Vice versa, this test may also be useful to determine the strength of an aversion. The consumer demand test is based on the concept to “work” for access to a preferred good or for avoiding an aversive stimulus. In experimental consumer demand tests animals have to pay a certain price to obtain a good. This can be realized by introducing a workload or obstacles that has to be overcome. Work can be implemented, for example, by pressing a lever or a switch [50,61], or by an obstacle like water or an adjustable weight barrier [62,63].

**Figure 1:**
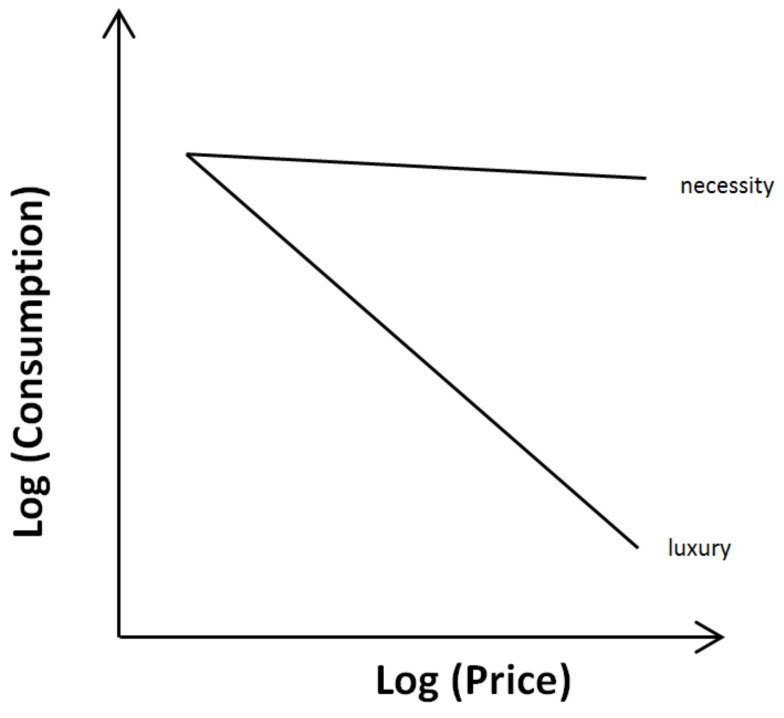
Consumer demand curves. The consumption is based on the actual demand and price. While necessities are consumed to a considerable extent regardless of price, luxury goods can easily be dispensed with, if the price becomes too high.

The derived data can be illustrated as a consumer demand curve with the specified price on the x-axis and the amount consumed on the y-axis (Figure 1). Consumer demand theory predicts that the amount consumed is negatively affected by the price. However, the range of change is influenced by the value of the respective good. For necessary goods, price increases have only a minor effect on the quantity of goods consumed, while for luxury goods, price increases affect largely the consumed quantity. With regard to animal welfare, particular emphasis is placed on the ultimate needs necessary for survival and reproduction. In the language of consumer demand, the ultimate needs would be similar to necessities, with the animal willing to pay almost any price to get this good. On the other hand, lower consumption of a good when the price is raised indicates that such a good is less valued and reflects a luxury, which is less important with regard to animal welfare [26,64]. Importantly, Dawkins pointed out that needs without an obvious influence on survival could still be of significant value to the individual animal [48,64]. As an example, she mentioned a caged bird, whose free-living conspecifics migrate in autumn. In free-living birds, migration increases survival, whereas a caged bird does not need to migrate to survive because it is sufficiently supplied. Nevertheless, the evolutionary developed urge to migrate may be that strong as to cause suffering if the behavior cannot be performed.

By training the animals to work for the access to certain goods, the preferential strength and the grade of necessity of this good can be determined by increasing the price. Therefore, consumer demand testing is a useful method for animal welfare research and severity assessment [45,48,64].

Sherwin used the consumer demand test to demonstrate the strength of preference for a running wheel or additional space in mice. The mice had to learn to press a switch several times to gain access to a running wheel, an extended tunnel or a complex tunnel system [65]. With increasing costs, the number of visits to the two tunnel systems decreased. However, the number of visits for the running wheel was unchanged. In the study by Lewejohann and Sachser, mice had to learn to press a lever to access an enriched cage. The mice pressed the lever up to 16 times, showing a high willingness to work for an enriched cage [50].

In order to use the consumer demand test, it has to be noted that the willingness of the animals to work depends on whether or not an adequate alternative is available. In addition, it has to be ensured that the animals have indeed learned how to get the goods, and a decrease in consumption is not due to deficits in associative learning. Notably, the animals have to be trained sufficiently to press a lever or a switch, and this training itself can be very time consuming. The animals are often placed in a separate cage so that they are trained and tested outside their familiar environment. It is also important to consider whether or not the animals are trained and tested during their active phase as this has a profound influence on the motivation for training and testing. Overall, a home cage-based test environment would be preferable as the animals could perform the training phase and consumer demand test during their active phase and in their familiar environment. This in turn could reduce many factors that might negatively affect the data.

## 5. Cognitive Bias Test

Recently, the cognitive bias test has been developed that promises to be a suitable method for animal welfare research and severity assessment. This test is also a test that allows examining the animal’s perspective. In brief, the test investigates the influence of previous experiences on the expectation of future events. Humans and also animals which have experienced negative events tend to have a “pessimistic” expectation regarding future events, meaning they expect additional negative events and react more hesitantly towards new situations. On the other hand, humans and animals are “optimistic” towards future events, if they had more positive experiences or are less worried [66]. The cognitive bias test thus reflects the current emotional state of an individual. Determining the emotional state of laboratory animals can contribute to the improvement of housing and testing conditions. This can lead to more valid data, which also might lead to better transferability of the results.

The emotional state is influenced by cognitive processes and, conversely, the emotional state influences cognitive processes [66,67]. Cognitive abilities enable humans and animals to orient themselves and adapt to their environment. Via a combination of cognition and the emotional components, information is collected and memorized with regard to its valence. This relationship is taken advantage of using the cognitive bias test.

**Figure 2:**
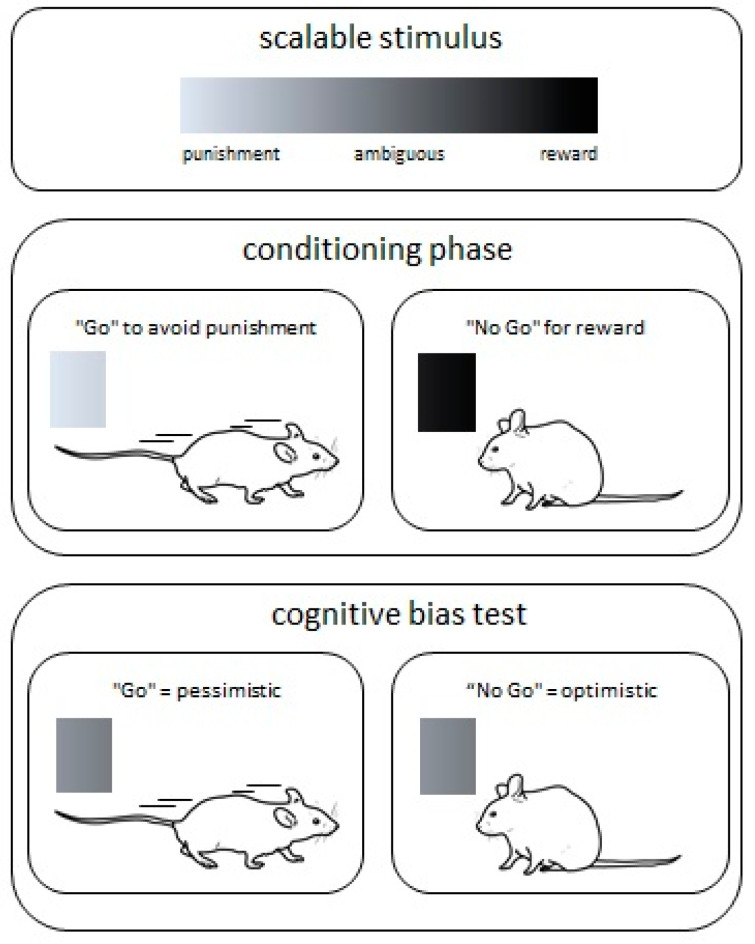
Cognitive bias test. During the conditioning phase the animals learn that one stimulus is associated with a punishment while the other stimulus is associated with a reward. In this Go/No Go example, the animals have to actively avoid a punishment (“Go”) or stay (“No Go”) to receive a reward. After successful conditioning, an ambiguous stimulus, which is calibrated in the middle of the scale between the positive and negative stimuli, is presented to test the cognitive bias. A “Go” behavior is interpreted as “pessimistic” and a “No Go” behavior as an “optimistic” emotional state.

So far, a number of different cognitive bias tests have been presented for different species such as rats, mice, horses, sheep, or honey bees [68,69,70,71,72,73]. The tests follow the principle of conditioning animals for scalable stimuli like tones or colors (Figure 2). Animals must learn that they receive a reward (e.g., tasty food) for the stimulus at one end of the scale and that they receive a punishment (e.g., air puff) for the other stimulus on the other end of the scale. After the conditioning phase the animals are exposed to experiences potentially influencing their emotional state. Such conditions may be changes in their home cage environment or experiences due to animal experimentation. Thereafter the actual cognitive bias test follows. For this test an ambiguous stimulus, which is calibrated in the middle of the scale between the positive and negative stimuli, is presented and the reaction toward this ambiguous stimulus is measured. If the response to the ambiguous stimulus is fast, the animal seems to anticipate a reward. This behavior is interpreted as an “optimistic” emotional state. If the animal does not response or the response to the ambiguous stimulus is rather reserved, the animal’s behavior is interpreted as “pessimistic”. This in turn indicates that the animal expects a punishment and the recent experiences seem to have had a negative influence on the emotional state.

One often-discussed aspect of the cognitive bias test is whether the test should be carried out according to a Go/No Go or a Go/Go principle, whereby “Go” would require an animal to actively reaching out (e.g., moving towards/away a reward/punishment) and “No Go” would require the animal to passively wait to receive a reward or avoid any action in order to be spared from punishment. Jones and colleagues found that rats reached the learning criterion when they had to actively approach a reward and passively avoid a punishment (i.e., “Go/No Go”). Vice versa, the learning criterion was not reached when applying a paradigm with “Go” to avoid punishment and “No Go” to receive a reward. Interestingly, mice behaved differently. Mice reached the learning criterion for the “Go” to avoid a punishment and “No Go” to receive a reward principle [74]. However, it is discussed whether the “No Go” behavior is less influenced by a negative emotional state, but rather by a lower motivation in general [75,76].

In addition, conclusions about the emotional state or “optimistic”/“pessimistic” behavior have to be made carefully as other factors might influence the animal’s behavior. For example, animals which receive an air puff as a punishment for the negative stimulus could become accustomed to it and, as a result, might show less avoidance behavior and react as they would for the rewarding stimulus. This would lead to results indicating a more optimistic behavior, although the cause would not be a positive experience, which such an experiment was meant to evaluate. Moreover, it is also possible that animals which experience a negative situation could perceive the ending of this situation as positive. In the final test (after the negative situation) the results then would also indicate optimistic behavior although the situation itself was negative. Thus, cognitive bias tests always have to be interpreted cautiously and in relation to the context. More information about critical methodological aspects of the cognitive bias test can be found in the reviews of Bethell, Gygax, or Roelofs [77,78,79].

The cognitive bias test has already been used to examine the influence of housing conditions on the emotional state. Harding and colleagues developed the first cognitive bias test, and conditioned rats to either press or not press a lever when hearing various tones. Rats which were housed under aversive unpredictable housing conditions (e.g., reversing dark/light cycle, damped bedding) pressed the lever less often than rats which were housed under normal conditions. This response to the ambiguous stimulus was interpreted as “pessimistic” and showed that unpredictable housing conditions had a negative influence on the emotional state of rats [68].

In another test, rats had to associate grades of sandpaper (fine or rough) with reward or punishment. The data indicated that rats which were housed first in standard cages without enrichment and then transferred to enriched cages showed an “optimistic” bias compared to rats which were housed permanently in non-enriched cages [80]. Similar results were given in a study with a depression-like phenotype in rats. Rats which were housed unenriched and then transferred to enriched cages showed a shift to an “optimistic” bias [69].

The first cognitive bias test for mice was developed by Boleij and colleagues in 2012. The mice were conditioned to various odor cues [70]. A spatial cognitive bias test for mice was developed by Kloke and colleagues in 2014 [81], showing that mice lacking a functional serotonin transporter tended to be more pessimistic compared to wild type mice.

Past studies have shown that the cognitive bias test is a useful method to examine the emotional state and the expectation regarding future events in animals. Therefore, this test also seems to be a suitable method for animal welfare research and severity assessment. The cognitive bias test can also be used to evaluate housing and experimental conditions of laboratory animals, and allows the animal’s point of view to be taken into account for adaptation, refinement and improvement. However, there are also disadvantages within the previous approaches. For example, in the above mentioned test designs, the actual test run could only be carried out once while training proved to be very time-consuming. Therefore, an automated touchscreen-based test design was developed [82]. As more trials per session can be performed in an automated test, the number of ambiguous trials per session can be better balanced. This is important to prevent the animals from learning that there is no reward or punishment for ambiguous stimuli [79,83], and it is possible to repeat the cognitive bias test. In addition, automated data collection avoids an observer bias, allowing neutral data evaluation [84]. However, in this touchscreen-based approach it is still necessary to place the animals in a separate test apparatus for training and testing. Therefore, an automated and home cage-based cognitive bias test would be of great advantage. Both the test itself and the conditioning could be carried out without the influence of handling, during the active phase of the animals and in their familiar environment. This would reduce external influences which could affect the cognitive bias of the animals.

## 6. Conclusions

The number of animals used for experimental purposes is still alarmingly high. This becomes particularly clear when surplus animals are counted in addition to the pure laboratory animal numbers [85]. It is therefore imperative for all researchers that 3R measures must continue to be used to further reduce these figures. With regard to animal welfare, all animals under human supervision must be taken into account. Especially for all surplus animals, animal welfare can sometimes be improved more easily [3].

Just as researchers can ask themselves what it takes to live a good life, subjective feelings are also of great importance for animals. Subjective experiences are linked to the behavior and physiology of the animal and should not be considered separately [86]. Sandøe states that well-being cannot be assessed by scientific methods alone. He therefore suggests that animal researchers and philosophers should work together to define and evaluate well-being [86].

In the case of animal experiments, animal welfare must be a top priority in addition to the scientific objective. It is therefore essential to assess the severity of procedures involving laboratory animals as objectively and accurately as possible. However, depending on the nature of the experimental and husbandry conditions, pain, suffering, or harm might be subtle and thus not easy to quantify.

Under laboratory and experimental conditions, animals are restricted in the development of their natural behavioral repertoire, and a wide range of husbandry conditions can be improved to refine the welfare of laboratory animals [3]. Animal experimental research basically involves procedures that, depending on the experiment, are associated with more or less pain, suffering or damage. Therefore, all procedures should be continuously examined for refinement possibilities to minimize suffering. Indeed, continuous monitoring of the health status of laboratory animals can make a huge contribution to reducing animal suffering [19,30,33,34,35,36]. All in all, there is still much room for improvement in the welfare of laboratory animals for those animal experiments that cannot be replaced in the foreseeable future. This certainly also includes promoting positive animal welfare in laboratory animals [10] rather than merely avoiding negative impacts.

Improving animal welfare requires methods to assess severity, of which several major approaches are discussed in this article. In addition to objectively measurable parameters, the animals’ perspective must be taken into account. Science can only indirectly ask the animals what they want or do not want (preference tests) or how much they want or do not want (consumer demand tests) certain goods. Science can also ask the animals only indirectly how their emotional status is within or after a specific situation (cognitive bias test). However, these approaches offer the possibility to better understand laboratory animals in their entirety, which can also lead to better animal research and results as there is growing evidence that impaired well-being affects the quality of data collected in animal studies [87].
